# *In Vivo* Manipulation of γ9^+^ T Cells in the Common Marmoset (*Callithrix Jacchus*) with Phosphoantigen and Effect on the Progression of Respiratory Melioidosis

**DOI:** 10.1371/journal.pone.0074789

**Published:** 2013-09-30

**Authors:** Thomas R. Laws, Michelle Nelson, Cecile Bonnafous, Helene Sicard, Christopher Taylor, Francisco Javier Salguero, Timothy P. Atkins, Petra C. F. Oyston, Caroline A. Rowland

**Affiliations:** 1 Biomedical Sciences Dept, Defence Science and Technology laboratory (DSTL) Porton Down, Salisbury, United Kingdom; 2 Innate Pharma, Marseille, France; 3 Animal Health and Veterinary Laboratory Agency (AHVLA), Weybridge, United Kingdom; Tulane University School of Medicine, United States of America

## Abstract

*Burkholderia pseudomallei* is a dangerous human pathogen. Phosphoantigens specifically the target primate specific γ9^+^δ2^+^ T cells subset and some have been developed as potential immunotherapeutics. Previously, we demonstrated that, when stimulated with the phosphoantigen CHDMAPP, γ9^+^δ2^+^ T cells aid in the killing of intracellular *B. pseudomallei* bacteria. Moreover, we found that common marmoset (*Callithrix Jacchus*) γ9^+^ T cells increase in frequency and respond to the phosphoantigen CHDMAPP and/or *B. pseudomallei*, in combination with IL-2, in a similar manner to human γ9^+^δ2^+^ T cells. Here we evaluate the efficacy of the phosphoantigen CHDMAPP, in combination with IL-2, as a therapy against *B. pseudomallei* infection, *in vivo*. We found that the previous studies predicted the *in vivo* responsiveness of γ9^+^ T cells to the CHDMAPP+IL-2 treatment and significant expansion of the numbers of peripheral and splenic γ9^+^ T cells were observed. This effect was similar to those reported in other primate species treated with phosphoantigen. Furthermore, splenocytes were retrieved 7 days post onset of treatment, restimulated with CHDMAPP or heat-killed *B. pseudomallei* and the cultured γ9^+^ T cells demonstrated no reduction in IFN-γ response when CHDMAPP+IL-2 animals were compared to IL-2 only treated animals. Using an established model of *B. pseudomallei* infection in the marmoset, we assessed the potential for using phosphoantigen as a novel immunotherapy. The CHDMAPP treatment regime had no effect on the progression of respiratory melioidosis and this was despite the presence of elevated numbers of γ9^+^ T cells in the spleen, liver and lung and an increased proportion of IFN-γ^+^ cells in response to infection. We therefore report that the common marmoset has proven a good model for studying the effect *in vivo* of γ9^+^ T cell stimulation; however, γ9^+^ T cells have little or no effect on the progression of lethal, respiratory *B. pseudomallei* infection.

## Introduction

γ9^+^δ2^+^ T cells are a T cell subset that is exclusive to primate species, which constitute a substantial proportion of total circulating T cells [1]. γ9^+^δ2^+^ T cells respond and increase in frequency specifically to a group of compounds referred to as phosphoantigens. Phosphoantigens are a secondary metabolite of most bacterial species [2]. Such compounds include the bacterial phosphoantigen (e)-4-hydroxy-3-methyl-but-2-enyl pyrophosphophate (HMBPP) and human isopentenyl pyrophosphate (IPP). It is known that γ9^+^δ2^+^ T cells increase in frequency during a variety of human infections with intracellular pathogens. These include: *Mycobacterium tuberculosis* [3], *Brucella suis* [4], *Francisella tularensis* [5] and *Listeria monocytogenes* [6]. This expansion of numbers suggests a role for γ9^+^δ2^+^ T cells in host-defence and *in vitro* studies have provided an insight into their potential anti-microbial effects. Using *in vitro* assays, γ9^+^δ2^+^ T cells have been reported to reduce the viability of several intracellular bacteria, including: *B. suis*, [7], *M. tuberculosis* [3] and *F. tularensis* [8]. Also, γ9^+^δ2^+^ T cells have been demonstrated to induce cytotoxicity of influenza infected macrophages [9] and inhibiting HCV replication [10] *in vitro*.

When considering the capacity for γ9^+^δ2^+^ T cells to limit infection *in vitro*, and that they increase in frequency during infection *in vivo*, there has been interest in using these cells as targets for immunotherapy for infectious diseases. For this reason, phosphoantigens have been developed as potential therapeutics for tumours and infectious diseases. One such phosphoantigen IPH1101 has been through Phase IIa clinical trials showing positive results for the treatment of Hepatitis C virus (HCV) [11]. IPH1101 and other HMBPP-based compounds, when used in combination with interleukin-2 (IL-2) can affect substantial increases in γ9^+^δ2^+^ T cells [12]. The critical difficulty with *in vivo* assessment of the efficacy of phosphoantigens is that this cell subset is exclusive to primate species. This has necessitated the use of non-human primate (NHP) models. The role of γ9^+^δ2^+^ T cells has been investigated in a number of NHP models of infectious disease and these include tuberculosis [13] and monkeypox infection [14]. Furthermore, the proliferative responses of γ9^+^δ2^+^ T cells to phosphoantigen have been measured in NHP models including the “Old World” primates: cynomolgus macaques [12] rhesus macaques [15], [16] and baboons [12]. Increases of γ9^+^δ2^+^ T cell numbers in these animals occurred within the first few days of treatment and was observed in both the blood and in the tissues including the lung [16]. The effect of HMBPP+IL-2 treatment has been investigated in two NHP infection models. In chronic HIV infection, HMBPP+IL-2 treatment leads to enhanced anti-viral immune responses [15]. In acute pulmonary *Yersinia pestis* infection, HMBPP+IL-2 treatment had no effect on survival; however, a marked reduction in immune pathology was observed in the organs of treated animals [17].

The common marmoset (*Callithrix jacchus*) has been used as the host in the development of a variety of highly virulent, potential bioweapon diseases, including: anthrax [18], tularemia [19], [20], marburg fever [21] and melioidosis [22]. Melioidosis is caused by *Burkholderia pseudomallei*, a Gram negative, intracellular, bacterial pathogen, which is a endemic to regions of Thailand, Indonesia and Australia [23], [24]. *B. pseudomallei* infection is especially acute following infection by the respiratory route. In cases of near-drowning or following tsunami, where infection by the aerosol route is suspected, symptoms of human Melioidosis are much more severe than other routes [25]. This is also observed using several animal models of infection including the mouse [26], [27] and the marmoset, where *B. pseudomallei* bacteria causes an acute systemic infection with animals surviving only a few days post infection following aerosol exposure [22]. Death in these animals is believed to be a consequence of severe pneumonia and respiratory failure. Most *B. pseudomallei* strains are resistant to antibiotics and, for this reason, therapy is aggressive and prolonged and relapse of the disease is frequent [28]. At present, no licensed vaccine is available to prevent the disease. Therefore, novel strategies for treating this infection are required. It is generally regarded that the infectious nature of *B. pseudomallei*, the lack of effective therapeutic treatments and its ability to survive as an aerosol all make the bacteria suitable for use as a biological weapon [29]. In previous *in vitro* studies, we have shown that γ9^+^ T cells from common marmoset increase in frequency, in response to the synthetic phosphoantigen CHDMAPP and this is comparable to the response of human γ9^+^δ2^+^ T cells [30]. In this study, we aimed to evaluate whether CHDMAPP (a synthetic phosphoantigen) in combination with IL-2 treatment could activate and increase numbers of marmoset γ9^+^ T cells *in vivo* and whether this might have therapeutic benefits against acute *B. pseudomallei* infection.

## Results

### CHDMAPP and IL-2 treatment has no adverse effects in common marmosets, *in vivo*

Animals were administered with one dose CHDMAPP and 5 daily doses of IL-2 (n = 4), IL-2 (n = 4) only or a PBS sham (n = 2) to assess the effects of these treatments on γ9 T cell responses. We determined that the CHDMAPP and IL-2 regime was well-tolerated. No aberration in body temperature or clinical signs and no adverse pathological effect were observed, in any animal, following histological exploration of lung, liver, kidney, spleen, lymph nodes (data not shown).

### The efficacy of CHDMAPP and IL-2 on γ9^+^ T cells in the common marmoset, *in vivo*

**Figure 1:**
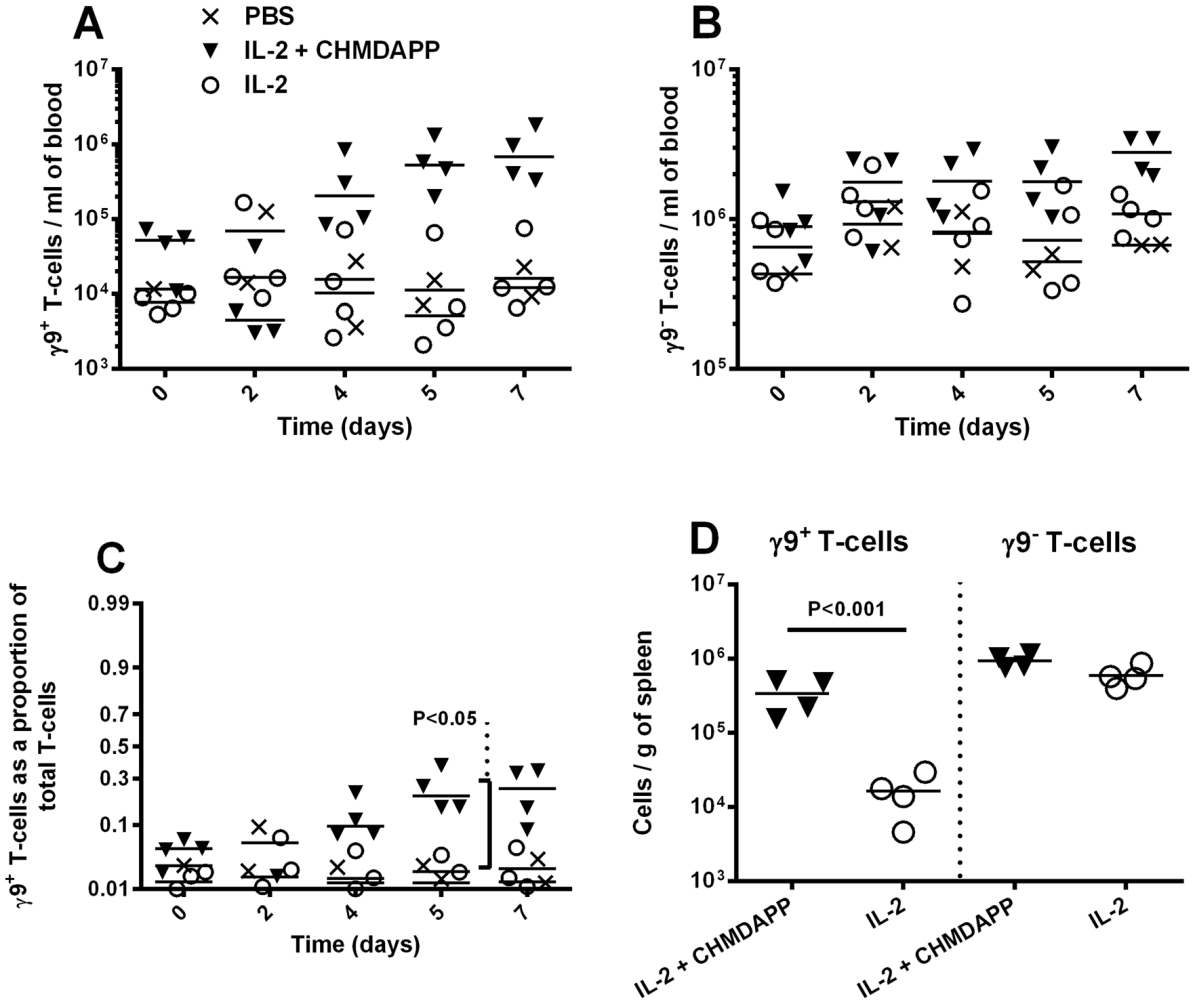
CHMDAPP^+^IL-2 causes *in vivo* expansion of γ9^+^ T cells in marmosets. Dynamics of marmoset γ9^+^ T cell numbers after treatment with CHMDAPP and IL-2 (n = 4), compared to a sham treated group (n = 2) and group treated with IL-2 only (n = 4). Animals either received a single dose of CHMDAPP (day 0, at 2.5 mg/kg) and five doses of IL-2 (days 0, 1, 2, 3, 4 and 5, at 0.18 U×10^6^ per kg) or only received the five doses of IL-2 or received PBS only. T cells, γ9^+^ and γ9^−^, were monitored in the blood at 0, 2, 4, 5 and 7 days post onset of therapy using flow cytometry. Panels A and B show the numbers of circulating γ9^+^ and γ9^–^ cells respectively. Panel C shows the same data expressed as the proportion of the T cells that are γ9^+^. Panel D shows the number of T cells, relative to mass, in the spleen of these animals at 7 days post onset of treatment. Each data point is a different animal at that time point. The lines are the medians and the P values are indicative of Bonferroni's post tests comparing parameters.

CHDMAPP and IL-2 treatment had a marked effect on the number of circulating γ9 T cells, in comparison to those treated with IL-2 only (Figure 1). Using a general linear model, we determined that CHDMAPP and IL-2 co-treatment had an overall effect on the number of γ9^+^ T cells (P<0.01, Figure 1A), but not γ9^−^ T cells (P>0.05, Figure 1B). We found that γ9^+^ T cell numbers rose to levels similar to those observed for all other γ9^−^ CD3^+^ T cells and peaked, at approximately 20%, 5–7 days post onset of treatment. A significantly greater proportion of γ9^+^ T cells were observed at day 5 in CHDMAPP+IL-2 treated animals in comparison with animals treated with IL-2 only (P<0.05, Figure 1C). 7 days following onset of treatment, we found a significant increase (greater than 10-fold) in γ9^+^ T cells in the spleens of animals treated with CHDMAPP+IL-2 co-treatment in comparison with IL-2 only treatment (P<0.001, Figure 1D).

**Figure 2:**
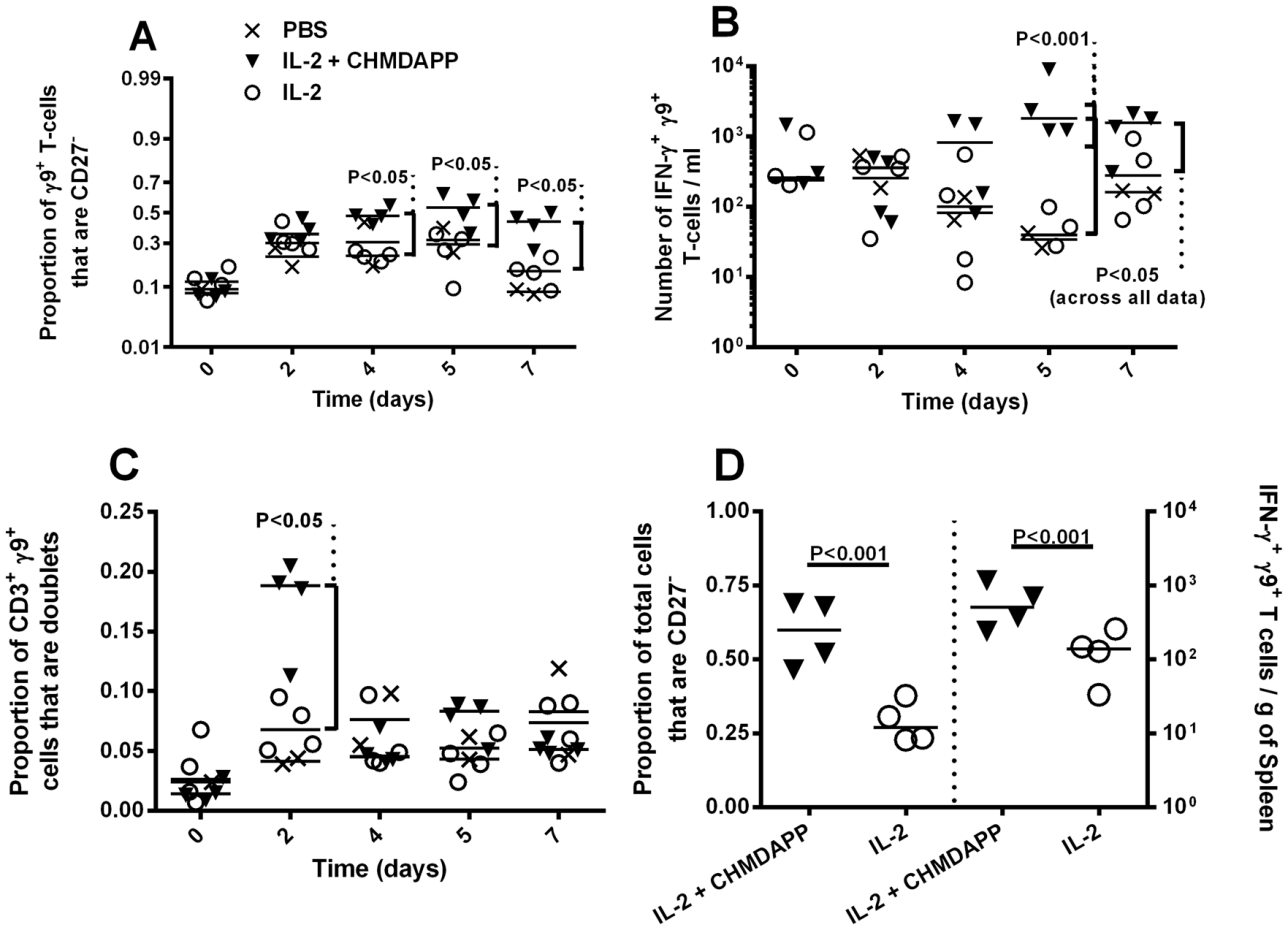
CHMDAPP with IL-2 alters the phenotype of γ9^+^ T cells by increasing the frequency of CD27^−^ γ9^+^ T cells and increasing the numbers of IFN^−^γ^+^γ9^+^ T cells. Phenotype of marmoset γ9^+^ T cells after treatment with CHMDAPP and IL-2 (n = 4), compared to a sham treated group (n = 2) and group treated with IL-2 only (n = 4). Animals either received a single dose of CHMDAPP (day 0, at 2.5 mg/kg) and five doses of IL-2 (days 0, 1, 2, 3, 4 and 5, at 0.18 U×10^6^ per kg) or only received the five doses of IL-2 or received PBS only. Panel A shows the proportion of circulating γ9^+^ cells not expressing CD27. Panel B shows the number of circulating γ9^+^ cells expressing IFN-γ. Panel C shows the proportion of circulating γ9^+^ T cells that register as clustered cells. Panel D shows CD27 and IFN-γ expression data generated from the spleen at 7 days post onset of treatment. Each data point is indicative of a single reading from and animal and the lines indicate the population medians. The lines are the medians and the P values are indicative of Bonferroni's post tests comparing parameters.

We also investigated the phenotype of circulating γ9^+^ T cells (Figure 2). We found that CHDMAPP and IL-2 co-treatment increased the proportion of γ9^+^ T cells that lacked CD27 expression (CD27^−^) (P<0.05 overall, Figure 2A). Additionally, using intracellular cytokine staining we determined that following CHDMAPP and IL-2 co-treatment a higher number of γ9^+^ T cells expressed IFN-γ in comparison with marmosets treated with IL-2 only (P<0.05, Figure 2B). However, this increase was not represented in the proportion of γ9^+^ T cells that were IFN-γ^+^ (data not shown), showing that this increase was proportional of the increase in total numbers of γ9^+^ T cells. Furthermore, we calculated the number of γ9^+^ T cells that were clustered cells (two or more cells attached to each other determined by event aspect ratio, by flow cytometry). We observed a sharp increase in the number of γ9^+^ clustered cells 2 days post CHDMAPP and IL-2 co-treatment in comparison with IL-2 treated animals (P<0.05, Figure 2C). CHDMAPP and IL-2 co-treatment had no effect on CD27, IFN-γ expression, or frequency of clustered cells, on γ9^−^ T cells when compared to IL-2 only treated animals (P>0.05, data not shown). Similar results were observed in the spleen 7 days post onset of treatment, where numbers of IFN-γ^+^ γ9^+^ T cells were higher in CHDMAPP and IL-2 co-treated marmosets (P<0.05) and an increase in the proportion of CD27- γ9^+^ T cells in CHDMAPP treated animals was also observed (P<0.001) when compared to their IL-2 only treated counterparts. This demonstrated systemic action of the treatment.

**Figure 3:**
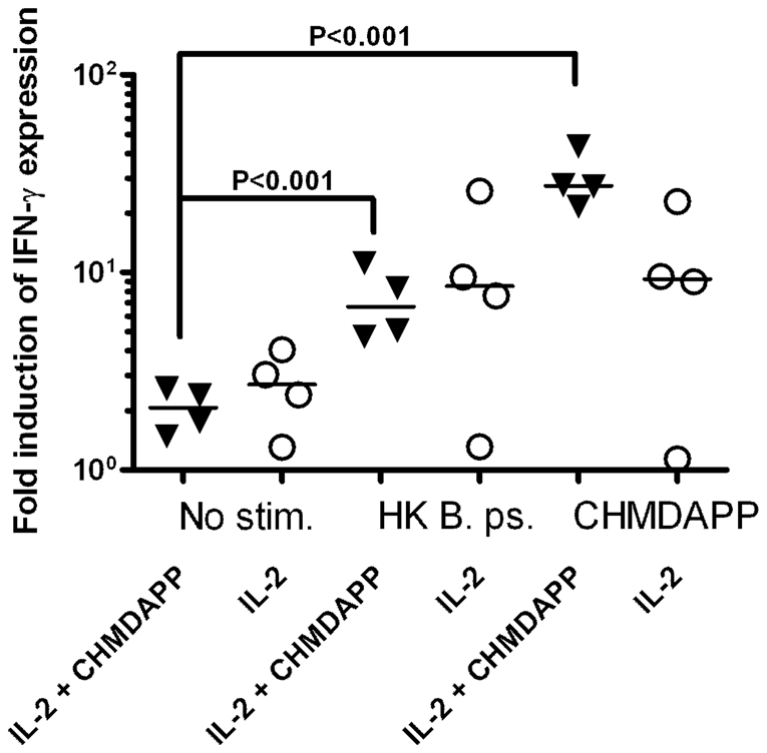
γ9^+^ T cells respond to restimulation with Heat-killed *B. pseudomallei* and CHMDAPP *ex vivo*. The effect of re-stimulation by CHMDAPP or heat-killed *B. pseudomallei* (HK B.ps.) on IFN-γ expression by γ9^+^ T-cells in cultured splenocytes. Fresh splenocytes were isolated from marmosets treated with CHMDAPP and IL-2 (n = 4), and group treated with IL-2 only (n = 4). Animals either received a single dose of CHMDAPP (day 0, at 2.5 mg/kg) and five doses of IL-2 (days 0, 1, 2, 3, 4 and 5, at 0.18 U×10^6^ per kg) or only received the five doses of IL-2 or received PBS only. These were cultured for 6 h with each stimulant and monitored for IFN-γ expression by flowcytometry. Fold induction was calculated by dividing the proportion of IFN-γ^+^ events at time of isolation by the proportion of IFN-γ^+^ events after *ex vivo* restimulation. Each data point represents the mean of 4 technical replicates and the line is the median of the 4 different animals. The lines are the medians and the P values are indicative of Bonferroni's post tests comparing parameters.

In order to verify the responsiveness of these γ9^+^ T cells, splenocytes were taken and subjected to further stimuli, *ex vivo* (Figure 3). We found increased intracellular IFN-γ expression in γ9 T cells from both CHDMAPP + IL-2 co-treated animals and IL-2 only treated animals in response to either CHDMAPP or heat-killed *B. pseudomallei* (P<0.05, to calculate the fold induction, the frequency of IFN-γ^+^ events were standardised to number of events determined prior to culture). Furthermore, we observed an increase in intracellular IFN-γ expression in γ9 T cells from IL-2 only treated marmosets (P<0.05). We found no evidence to indicate that the *in vivo* treatment of the animals had any effect on their splenic γ9^+^ T cells responses *ex vivo* after stimulation (P>0.05); although, due to the low numbers of γ9^+^ cells in the splenic cultures of animals treated with IL-2 only, high variation was observed making the statistical power of this comparison low.

In summary, we found that the CHDMAPP and IL-2 treatment stimulated γ9^+^ T cells to increase in number and activation state. This was specific to the γ9^+^ T cell population and numbers and activation of γ9^−^ T cells remained unchanged.

### The effect of CHDMAPP on the progression of respiratory melioidosis in marmosets

**Figure 4:**
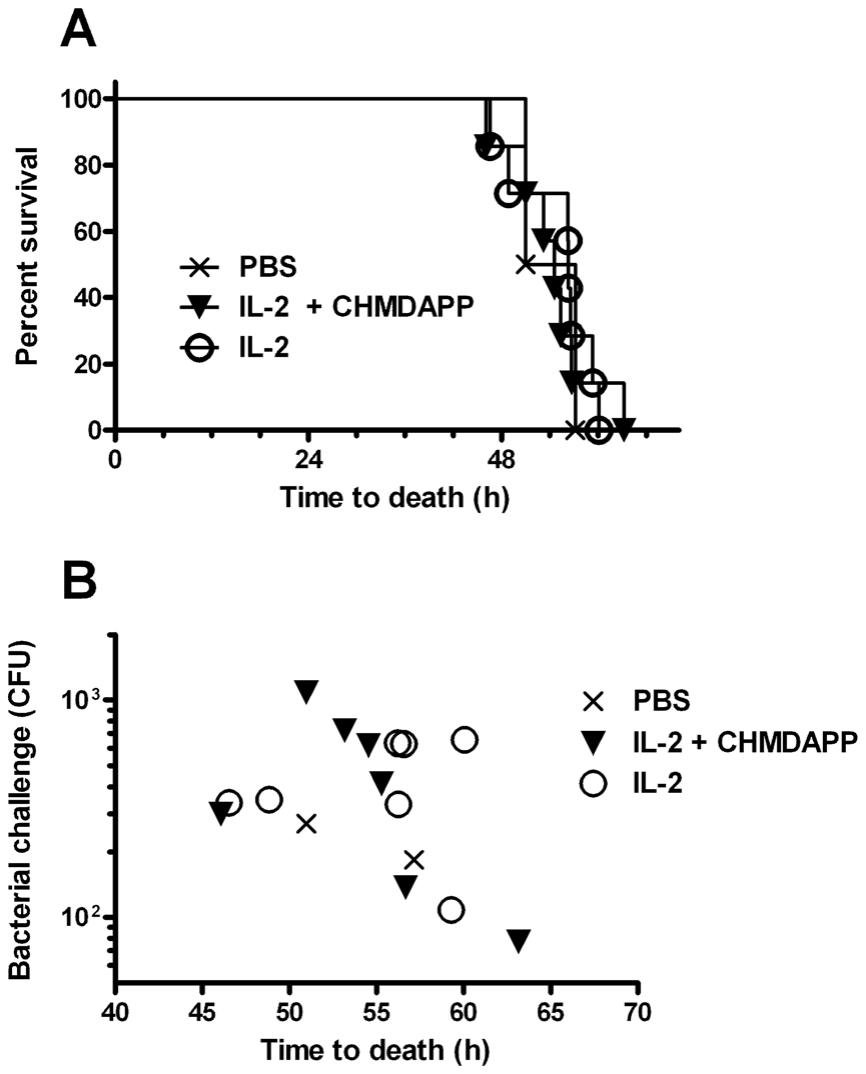
Treatment with CHMDAPP+IL-2 does not affect survival of marmosets infected with an aerosol challenge of *B. pseudomallei*. Survival of marmosets treated with CHMDAPP and IL-2 (n = 7), and group treated with IL-2 only (n = 7). Animals received a single dose of CHMDAPP (day 0, at 2.5 mg/kg) and five doses of IL-2 (days 0, 1, 2, 3, 4 and 5, at 0.18 U×10^6^ per kg) or only received the five doses of IL-2 or received PBS only. Animals were then challenged with 77–1,081 CFU of *B. pseudomallei* strain K96423 at day 5 post onset of treatment and survival after infection was monitored using lethal endpoints. Panel A shows a Kaplan Meier plot of survival. Panel B shows the relationship between challenge dose and survival time.

Marmosets were treated with the same regimen of IL-2 and CHDMAPP as used in the previous study (one dose CHDMAPP and 5 daily doses of IL-2 (n = 7), IL-2 (n = 7) only or a PBS sham (n = 2)). On the 5^th^ day animals were challenged with *B. pseudomallei* by the aerosol route. Individual marmosets received individual calculated challenges of bacteria that ranged from 77 up to 1,081 CFU. Analysis of these challenge doses demonstrated that they were evenly distributed between treatment groups. We found that the CHDMAPP and IL-2 co-treatment had no effect on survival when compared to the IL-2 only treated group (Figure 4A). It is known that bacterial challenge dose can affect the rate of disease progression in this model of melioidosis [22]. For this reason we also investigated the treatment based effects, on survival, using initial challenge dose as a covariate and confirmed that there was no evidence that treatment affected survival time (P>0.05) (Figure 4B).

**Figure 5:**
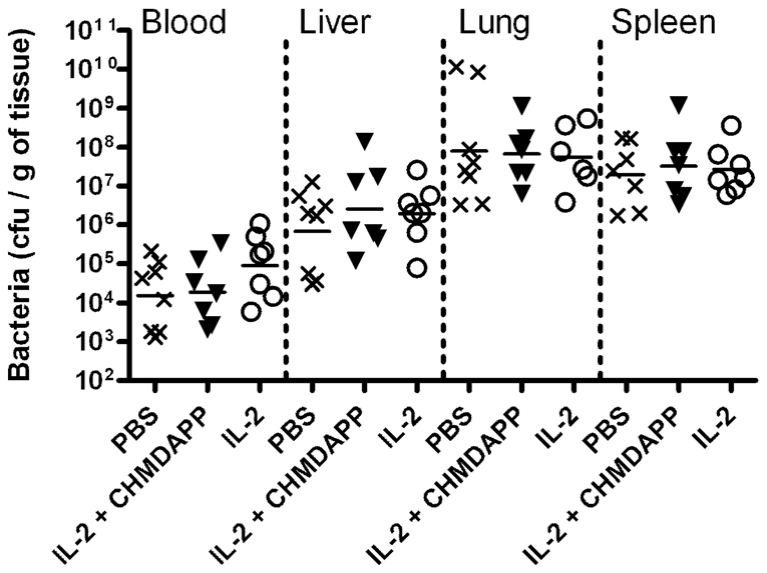
Treatment with CHMDAPP+IL-2 does not affect bacterial colonisation in marmoset tissues following aerosol challenge with *B. pseudomallei*. Bacterial colonisation in marmoset tissues treated with CHMDAPP and IL-2 (n = 7), and group treated with IL-2 only (n = 7). Animals received a single dose of CHMDAPP (day 0, at 2.5 mg/kg) and five doses of IL-2 (days 0, 1, 2, 3, 4 and 5, at 0.18 U×10^6^ per kg) or only received the five doses of IL-2 or received PBS only. Animals were then challenged with 77–1,081 CFU of *B. pseudomallei* strain K96423 at day 5 post onset of treatment. The graph shows bacterial colonisation measured using standard viable counting. The sham group (PBS) includes some legacy data, from previously published experiments [22]. Each data point represents a reading of a single organ from a single marmoset and the line represents the population median.

*B. pseudomallei* infection in marmoset results in substantial bacterial burden in many tissues. In previous work, bacterial numbers in lung (the primary loci of infection) are relatively constant at terminal time points whereas high burdens are observed in secondary loci were infections have lasted longer due to lower challenge dose [22]. This indicates that disease in the lung is likely to be critical in this model of melioidosis. The bacterial colonisation in various tissues in marmosets was measured. We found that CHDMAPP and IL-2 co-treatment had no measurable effects on bacterial numbers, when compared to an IL-2 only treated group (Figure 5, P>0.05, in all organs). In order to verify the repeatability of our model and increase sample size, we have compared data generated in this study to data from previous studies where marmosets were challenged by the same route also with between 100 and 1,000 CFU. We observed no difference between current and this ‘legacy’ data.

### Immune responses in marmosets following CHDMAPP and IL-2 treatment and *B. pseudomallei* infection

**Figure 6:**
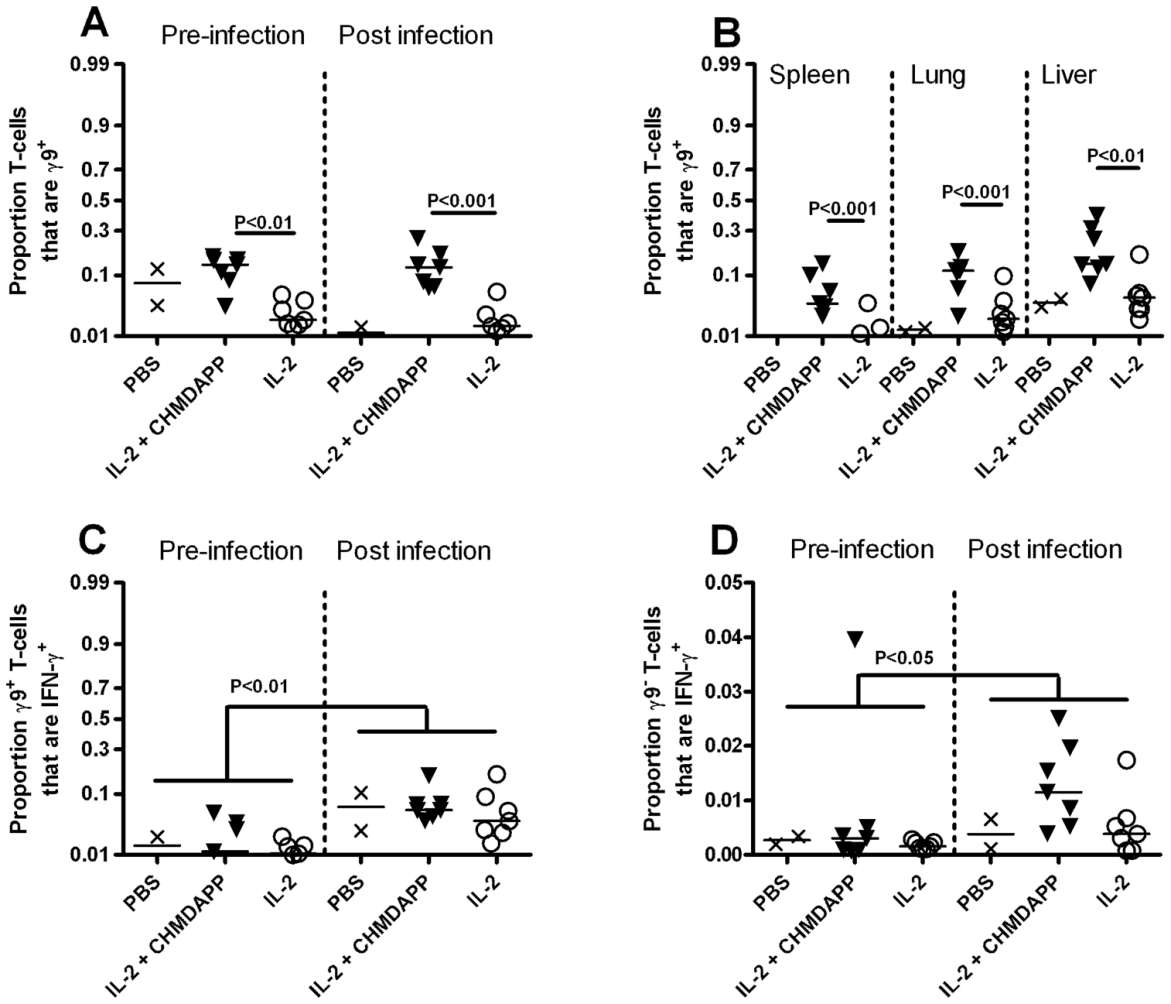
*B. pseudomallei* infection increases the proportion γ9^+^ T -**cells in CHMDAPP+IL-2 treated animals.** γ9^+^ T cell response in marmosets treated with CHMDAPP and IL-2 (n = 7), and group treated with IL-2 only (n = 7). Animals received a single dose of CHMDAPP (day 0, at 2.5 mg/kg) and five doses of IL-2 (days 0, 1, 2, 3, 4 and 5, at 0.18 U×10^6^ per kg) or only received the five doses of IL-2 or received PBS only. Animals were then challenged with 77–1,081 CFU of *B. pseudomallei* strain K96423 at day 5 post onset of treatment. Panel A shows the proportion of T cells expressing the γ9 receptor 1 day pre infection and post mortem, in the blood. Panel B shows the shows the proportion of T cells expressing the γ9 receptor in the organs. Panel C and D show the proportion of γ9^+^ and γ9^−^ T cells expressing IFN-γ respectively. Each data point represents a reading of a single organ/blood sample from a single marmoset and the line represents the population median. P values are indicative of Bonferroni's post tests comparing parameters.

Immunological parameters that were previously measured without infection were also measured in the infection experiment, using a pre-infection bleed and tissues and blood post-mortem (Figure 6). We found that, compared to the IL-2 only treated group, CHDMAPP and IL-2 co-treatment significantly increased the proportion of circulating T cells that were γ9^+^ pre-infection (P<0.01) and post infection (P<0.001) (Figure 6A). This was also true for the spleen, lung and liver (all P<0.01, Figure 6B). While the total number of IFN-γ^+^ γ9^+^ T cells increased in line with the numbers of γ9^+^ T cells following CHDMAPP and IL-2 co-treatment (consistent with the previous ‘efficacy’ experiment), the proportion γ9^+^ T cells expressing IFN-γ remained constant (P>0.05, Figure 6C). Infection increased the proportion of γ9^+^ T cells expressing IFN-γ (P<0.001) which was not affected by different treatment regimens (P>0.05, Figure 6C). We also found that the infection increased the number of γ9^−^ T cells expressing IFN-γ (P<0.05, Figure 6D). Additionally, when compared to the IL-2 only treated group, γ9^−^ CD3^+^ T cells in the blood of CHDMAPP and IL-2 co-treated animals post mortem had increased IFN-γ expression (P<0.05, Figure 6D). The expression of CD27 was also measured on T cells; however no measurable changes in its expression, on γ9^+^ T cells or γ9^−^ CD3^+^ T cells, was observed (P>0.05). A number of cytokines were investigated in post-mortem samples (data not shown). While we found that the treatments had no measureable effect on cytokine concentration (P>0.05, in all cases), production of IFN-γ, IL-6, IL-1β, MCP-1 and TGF-β was detected in all of the infected tissues (blood, liver lung and spleen). We also observed small amounts of IL-10 in the spleen and liver.

### Pathological assessment of tissues and correlates to other measured parameters in marmosets post CHDMAPP and IL-2 treatment and *B. pseudomallei* infection

**Figure 7:**
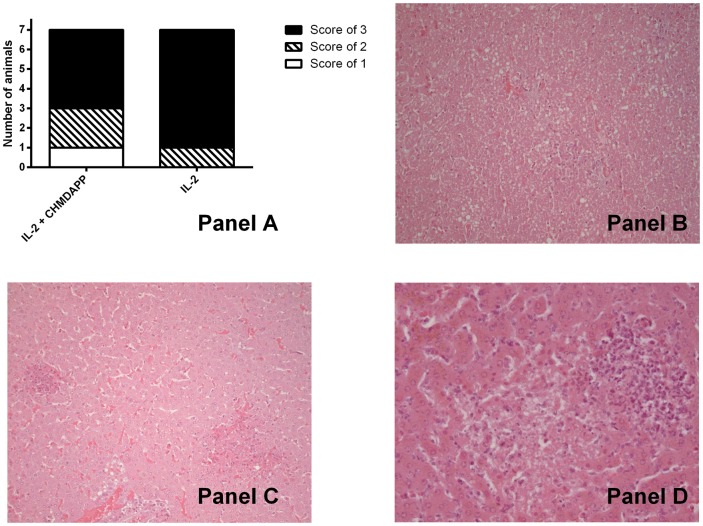
The liver of marmosets, some pre -**treated with the immuno**-**stimulant CHMDAPP and IL-2 after infection with an aerosol challenge of** ***B. pseudomallei*** **show a variety of levels of pathology.** Liver pathology was measured in animals treated with CHMDAPP and IL-2 (n = 7), and group treated with IL-2 only (n = 7) using samples take post lethal endpoint. Animals received a single dose of CHMDAPP (day 0, at 2.5 mg/kg) and five doses of IL-2 (days 0, 1, 2, 3, 4 and 5, at 0.18 U×10^6^ per kg) or only received the five doses of IL-2 or received PBS only. Animals were then challenged with 77–1,081 CFU of *B. pseudomallei* strain K96423 at day 5 post onset of treatment. Liver samples were investaged by tissue sectioning and H&E stain. Liver pathology in these animals was blindly set against a scale from 0 (no pathology) to 3 (severe pathology). Panel A shows a chart depicting the frequency of the different levels of liver pathology observed in this experiment. Panel B shows an example of liver pathology in an animal set a score of 1 (x200 magnification), panel C a score of 2 (x200 magnification) and panel D a score of 3 (x400 magnification).

Observed pathology in infected marmosets at the terminal time point was similar to that previously observed [22]. Pathology scores were generated for each organ from each individual animal. These scores consisted of a value of 0–3, where 0 indicated no visible pathology and 3 indicated severe pathology. All the lungs and spleens observed had a pathology score of 3. The liver displayed a range of values (1–3) (Figure 7). Of the 7 animals treated with IL-2 only, 6 animals had a liver pathology score of 3 and 1 had a score of 2. Of the 7 animals co-treated with CHDMAPP and IL-2, 1 animal had a score of 1, 2 animals a score of 2 and 4 animals had a score of 3. Both sham treated animals had liver pathology scores of 3. Non-parametric comparison of CHDMAPP and IL-2 co-treated animals in comparison with IL-2 only treated animals did not elude to a treatment-based decrease in liver pathology (P>0.05).

The infection study generated 43 metrics in which variation was observed. These included; time-to-death, pathology in the liver, estimated challenge dose, 6 cytokine concentrations across 4 locations (lung, liver, spleen and blood), proportions of γ9^+^ T cells across 4 locations, proportions of IFN-γ^+^ γ9^+^ T cells and γ9^−^ T cells across 4 locations, viable bacteria across 4 locations and bacterial genomes across 4 locations. A correlation matrix was generated from this data to further understand the relationships between these factors.

**Figure 8:**
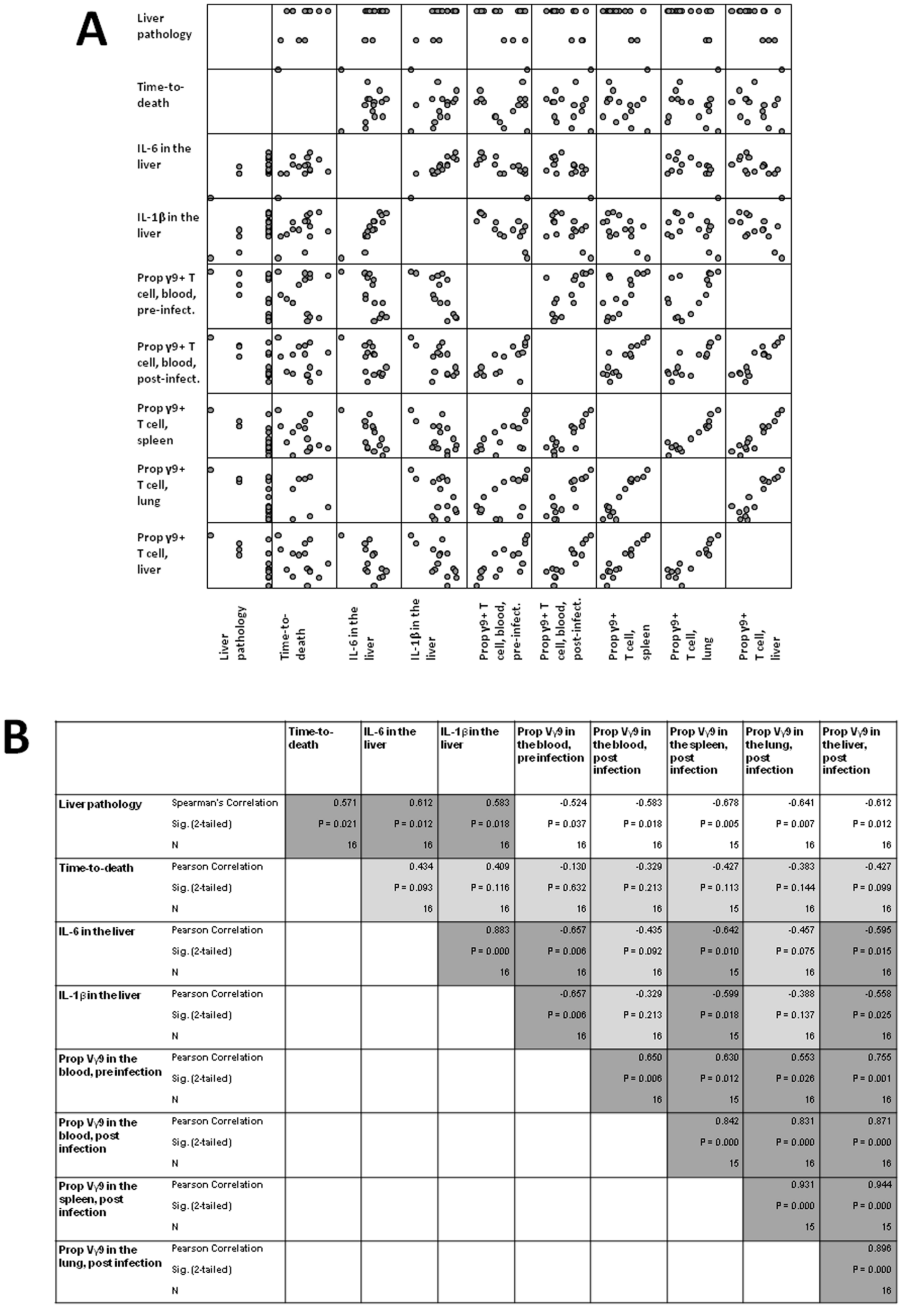
A variety of interactions between several parameters were observed in marmosets pre-treated with CHMDAPP+IL-2 and infected with *B. pseudomallei*. Animals were treated with CHMDAPP and IL-2 (n = 7), and group treated with IL-2 only (n = 7). Animals received a single dose of CHMDAPP (day 0, at 2.5 mg/kg) and five doses of IL-2 (days 0, 1, 2, 3, 4 and 5, at 0.18 U×10^6^ per kg) or only received the five doses of IL-2 or received PBS only. Animals were then challenged with 77–1,081 CFU of *B. pseudomallei* strain K96423 at day 5 post onset of treatment. At the lethal end point multiple parameters were read and correlation matrices of liver pathology scores, time-to-death, IL-6 concentration within the liver post mortem, IL-1β concentration within the liver post mortem, and proportion of γ9^+^ T cells in the blood pre and post mortem and in the liver, lung and spleen post mortem were performed. Panel A shows a matrix scatter plot showing the relationship between these parameters. Panel B shows the statistical analysis of these relationships. These two panels directly relate to each other. Pearson's correlations were performed with the exception of correlation to liver pathology scores, where the Spearman's method was used. White boxes indicate significant negative correlations, and dark grey boxes indicate significant positive correlations.

In short, we found that: as survival increased, liver pathology increased and higher liver pathology was associated with higher inflammatory cytokines in the liver and lower γ9^+^ T cells (Figure 8). Further analysis (Data S1 and Figure S1) also identified that: increased numbers of γ9^+^ T cells in the lung and liver are associated with higher numbers of IFN-γ^+^ other T cells. We found some evidence that the numbers of IFN-γ producing cells is negatively associated with anti-inflammatory cytokines; that γ9^+^ IFN-γ^+^ T cells were positively associated with blood inflammatory cytokines and that γ9^−^ IFN-γ^+^ T cells were positively associated with infectious load in the blood, liver and lung. We found that higher challenge doses were associated with numbers of γ9^−^ IFN-γ^+^ T cells prior to infection, splenic inflammatory cytokines and viable bacteria in the blood.

## Discussion

In this study, we aimed to determine the efficacy of phosphoantigen treatment against an acute bacterial infection, respiratory Melioidosis. We have shown that the marmoset is an appropriate model for investigating the *in vivo* responsiveness of γ9^+^ T cells to CHDMAPP+IL-2 with a significant increase in numbers occurring 5 days following treatment. This effect was observed both in the blood and also in the spleen. This response was predicted by previously published *in vitro* studies, these studies showing that marmoset γ9^+^ T cells increase in number and respond to the synthetic phosphoantigen CHDMAPP [31] in a similar manner to human γ9^+^δ2^+^ T cells [30]. CHDMAPP-mediated γ9^+^ T cell expansion in numbers, in marmosets was temporally similar to the increase in γ9^+^δ2^+^ T cell numbers observed in rhesus macaques to the bacterial phosphoantigen HMBPP+IL-2 [16] and cynomolgus macaques using the synthetic phosphoantigen Picostim+IL-2 [32]. CHDMAPP+IL-2 induced a population of CD27 negative γ9^+^ T cells in marmoset blood and spleen from day 4 following treatment. This is consistent with increases in CD27^−^CD45RA^−^ subset induced in other primate studies with HMBPP+IL-2 treatment *in vivo* between 4 and 7 days following dosing (15; 16). CD27^−^ γ9^+^δ2^+^ T cells were identified as functionally differentiated effector cells during *M. tuberculosis* infection [33] suggesting that the CD27 negative γ9^+^ T cell population generated following CHDMAPP treatment is an appropriate phenotype for treating intracellular bacterial infection.

IFN-γ is important for generating anti-microbial responses to intracellular bacterial pathogens including *B. pseudomallei* [34], *B. mallei* [35], *F. tularensis* [36], and *M. tuberculosis* [37]. A subpopulation of IFN-γ^+^ γ9^+^ T cells increased proportionally with the increase in total γ9^+^ T cells; in both the periphery and spleen. This suggests that the increase in IFN-γ^+^ numbers was due to the expansion of the frequency all γ9^+^ T cells (including the IFN-γ^+^ subset) by CHDMAPP+IL-2 treatment and not an increase in IFN-γ production by the γ9^+^ T cells. In addition, γ9^+^ T cells were able to further recognise and respond *ex vivo* to both CHDMAPP and heat-killed *B. pseudomallei ex vivo* following treatment with an IFN-γ effector response. This effect was also observed following restimulation with HMBPP in other NHP studies [16]. This presents us with an interesting question. *In vivo*, after the primary treatment with CHDMAPP+IL-2 we observed no increase in the proportion of IFN-γ^+^, only an increased frequency of all γ9^+^ T cells, which include the IFN-γ^+^ subset. Why then did we observe an increase in proportion of the γ9^+^ IFN-γ^+^ subset after *ex vivo*, secondary stimulation and not the primary, *in vivo* stimulation? This might be a function of the cells being cultured *ex vivo*. Alternatively, it is notable that no IL-2 was included in the *ex vivo* stimulation and this may have favoured stimulation of IFN-γ^+^ cells. Heat-killed *B. pseudomallei* has previously been shown to stimulate γ9^+^ T cell proliferation *in vitro* in a phosphate-dependent manner [30] suggesting that γ9^+^ T cells are responding to the bacterial phosphoantigen (HMBPP) component of the heat-killed bacteria. This is supported by the observed increase in IFN-γ producing γ9^+^ T cells in response to *B. pseudomallei in vivo* suggesting that γ9^+^ T cells were responding to the bacteria during infection leading to enhancement of effector responses. This is probably due to the HMBPP from *B. pseudomallei* further stimulating the γ9^+^ T cells *in vivo.* In addition to the effects on γ9^+^ T cell IFN-γ expression, an apparent ‘bystander’ effect on γ9^−^ T cells IFN-γ expression was observed during infection suggesting that the general T cell response was skewed towards an IFN-γ effector response appropriate for intracellular pathogen killing.

At 2 days post onset of treatment, prior to detection of increased CD27^−^ and IFN-γ^+^ γ9^+^ T cell phenotypes, the frequency of clustered γ9^+^ T cells significantly increased. We hypothesize that this is a consequence of cell division, as the γ9^+^ T cell population is beginning to expand at this time point. An alternative hypothesis is that an increased expression of adhesins on the cell surface may cause cell adhesion prior to migration to the tissues. γ9^+^ T cells are known to migrate from the periphery to the tissues including the lung following phosphoantigen treatment [16]. Measurement of cell-surface adhesion markers or levels of γ9^+^ T cells in the tissues to determine the nature of these clustered cells should be explored in future studies.

Overall, the marmoset was found to respond to CHDMAPP+IL-2 treatment in a comparable manner to other NHP species. Therefore, we examined the therapeutic potential of CHDMAPP+IL-2 against *B. pseudomallei* infection *in vivo*.

Following pulmonary exposure of marmosets to *B. pseudomallei,* an acute infection developed that was highly comparable to previous studies where we observed a substantial bacterial load in all animals post mortem [22]. A similar course of Melioidosis was observed in Rhesus macaques and African green monkeys [38] and in human Melioidosis; assumed cases of inhalational infection also have a high mortality rate with severe pneumonia developing within the first few days of infection [25].

Marmosets were given the validated treatment regimen and increased numbers of γ9 T cells was confirmed at day 4 post onset of treatment and prior to infection. Despite this, CHDMAPP+IL-2 had no discernable impact on animal survival or bacterial burden in any tissue following lethal (between 77 and 1,081 CFU) aerosol challenge with *B. pseudomallei*. Although studies have been performed to assess the effectiveness of phosphoantigen therapy during chronic infections including HIV [15] and monkeypox [14], there is only one published instance where phosphoantigen treatment of an acute infection in an experimental NHP model has been described. In Huang *et al* [13], macaques were infected with *Yersinia pestis* by the respiratory route to model pneumonic plague. Although, as in our study, Huang *et al* reported HMBPP and IL-2 treatment had little impact on survival; however, clear differences in lung pathology were observed, and the therapy was reported to have beneficial effects on the integrity of the lung. This improvement was associated with γ^+^δ^+^ T cell responses in the tissues.

Despite having no effect on survival, elevated levels of γ9^+^ T cells were found in CHDMAPP+IL-2 treated marmosets, at post-mortem, in blood, spleen, liver and lung demonstrating it had the desired effect on these cells which was maintained during infection. γ9^+^ T cell number expansion correlated well across tissues suggesting that variability in response was a consequence of inter-individual variation and not inter-tissue variation. γ9^+^ T cell number expansion also negatively correlated to liver pathology (which was the only organ to show variation in pathology, post-mortem) and some inflammatory cytokines in the liver. Additionally, we found that liver pathology correlated with time-to-death. Correlation does not assume causality and it is possible that either: [1] the marmosets that survived longer had longer had more time to accrue liver pathology, increase inflammatory response and a longer time for the expanded γ9^+^ T cell population to decay, or [2] the γ9^+^ T cell population has driven down the inflammatory cytokines, therefore decreasing pathology and increasing survival. It is unlikely that liver pathology contributes greatly to survival, in that death in this model appears to be caused by the primary pneumonia [22].

A massive pro-inflammatory response was mounted during *B. pseudomallei* infection in all the tissues and the treatment did not have any overall effect on this. The levels of IL-6 and IL-1β we observed were similar to those noted in other NHP species infected with *B. pseudomallei* [38]. Associations were observed between initial estimated dose and inflammatory cytokines in the spleen and viable bacterial numbers in the blood post mortem. This may suggest that higher doses of bacteria exacerbate the infection by inducing an inappropriate inflammatory response.

Huang *et al* described that a reduction in lung pathology corresponded with γ9 T cell accumulation and TGF-β in the lung [13]. We also observed evidence for this in that TGF-β correlated to the γ9^+^ T cell concentration, in the blood, prior to infection. Also, the concentration of γ9^+^ T cells expressing IFN-γ, post mortem, in the blood negatively correlated with IL-10 concentration in the liver, suggesting a possible negative interaction between γ9^+^ T cell activation and anti-inflammation in the liver. We found no evidence for an association between TGF-β and IL-10 concentration and γ9^+^ T cells in other tissues. We did, however, find evidence for a role of activated γ9^+^ T cells in the cytokine concentration in the blood, where proportion of γ9^+^ IFN-γ^+^ T cells correlated with inflammatory cytokine concentrations in the blood, post mortem. It is unclear whether this was an association or a cause. It appears from these correlations that γ9^+^ T cells maybe having differential effects in the tissues with both regulatory and pro-inflammatory roles.

In conclusion, we found that the marmoset is a relevant model for assessing the immunotherapeutic potential of phosphoantigens and for exploring the biology of γ9^+^ T cells *in vivo*. However, treatment with CHDMAPP+IL-2 had no large scale effects on the pathogenesis of pulmonary Melioidosis in this model. Our observations indicate that γ9^+^ T cells may be playing a role during infection. Due to the observed immune effects of this treatment it may be beneficial to optimise the phosphoantigen treatment regime in combination with antibiotics to improve bacterial clearance and the outcome of disease.

## Materials and Methods

### Animals husbandry, treatment and infection

All animal studies were carried out in accordance with the UK Animals (Scientific Procedures) Act of 1986 and the Codes of Practice for the Housing and Care of Animals used in Scientific Procedures 1989. The licence application underwent approval by the local ethical review process with the Dstl ERP before submission and approval with the UK Home Office and Animal Procedures Committee (an independent committee that offers advice to The Secretary of State of the ethics of the proposed work). The project licence that covers this work is 30/2300. Common marmosets (*C. jacchus*) were obtained from the Dstl Porton breeding colony as healthy sexually mature animals, aged between 36 and 60 months old and weighing between 320 and 480 g at the start of the experiment. Animals were housed in mixed sex pairs within the Experimental Animal House. Several weeks prior to the experiments marmosets were surgically implanted with telemetry devices. Pain relief (carprofen, a NSAID and antibiotics) was only provided during and for at least 5 days after surgical implantation of the telemetry devices. A minimum of 5 days prior to challenge animals were moved into the bio-containment facility and in specially designed cages housed within a Class 3 rigid walled isolator, with a maximum of four cages within an isolator. They were housed as female/vasectomised male pairs. Animals were feed once a day with primate pellets and a selection of fresh fruit, vegetables, hard boiled eggs, etc. and were provided fresh forage material and water was provided Ad-libitum. Environmental enrichment included a selection of wooden perches, ropes, buckets, Tupperware boxes and forage trays. Once febrile, animals were monitored at <4 h intervals remotely using cameras and telemetry, in order to minimise stress. A humane endpoint was used to alleviate unnecessary pain and suffering in the infection study and was determined by a greater than 1 degree drop in body temperature, following fever, associated with increasing clinical signs, typically lethargy, piloerection and a subdued nature [22]. At this time animals were sedated with ketamine and euthanized by an overdose of pentabarbitone.

Doses of IL-2 (Proleukin, Chiron, at 0.18 U×10^6^ per kg) and CHDMAPP (Innate Pharma, at 2.5 mg/kg) for individual marmosets were calculated by using the weight of the animals immediately prior to the experiment. Optimal dosing regimens for CHDMAPP and IL-2 had been established in previous studies [31]. Animals received 5 daily doses of IL-2 by the intra-peritoneal route in 100 μl of sterile phosphate buffered saline (PBS, Gibco™); control animals received PBS only. CHDMAPP-treated animals received 1 dose of CHDMAPP subcutaneously approximately 15 min prior to the IL-2 treatment on the first day of injections, in 100 μl of PBS. Infection studies were conducted as previously described [22].

### Sample processing and flow cytometry

At the end of the efficacy study, after terminal anaesthesia, only spleen and blood were taken for flow cytometric analysis and samples of lung, liver, spleen and kidney were taken for histopathology. At the end of the infection study, after terminal anaesthesia, samples of lung, liver, spleen, and blood were taken for both histopathology and flow cytometry. Sections of tissue were taken for histopathology and the remainder was weighed, removed aseptically and homogenized in 2 ml PBS using 20 μm cell strainers (Corning™, UK) for flow cytometry. For terminal phlebotomy, blood was removed by cardiac puncture and placed into citrate tubes (BD Biosciences™, UK). For phlebotomy of living animals, 50–300 μl of blood was removed from the femoral vein using a 1ml needle and placed into 2 ml tubes containing EDTA.

Samples (200 μl) of blood, spleen and lung homogenate were taken for immunological analysis. 1 μl of Golgi plug (BD Biosciences, UK) was added to each of these tubes (to a final concentration of 5 μl per ml) and they were incubated for 6 h in the efficacy study and 2 h in the infection experiment. These were centrifuged using a microfuge (300 g for 5 min) and the supernatant was taken and stored at −80°C for up to 6 months. For erythrocyte lysis, samples were resuspended in 1 ml of PharmLyse (BD Biosciences, UK) and incubated for 5 min at room temperature. Samples were centrifuged using a microfuge (300 g for 5 min) and resuspended in 200 μl of PBS with 2 μl of each of the following fluorochrome labelled antibodies: anti-CD27 (M-T271, PE), anti-CD3 (SP34–2, PerCP-cy5.5), and anti-γ9 (7B6, APC). All antibodies were commercially available (BD Biosciences, UK) with the exception of the anti-γ9 antibody that was a kind gift from Prof. Mark Bonneville (INSERM). Samples were incubated for 15 min at room temperature and 16% paraformaldehyde was added to make a final concentration of 4%. Samples were incubated for 24 h at 4°C and intracellular cytokine staining was performed as described in manufacturer's instructions (Cytofix Perm/wash step, BD Biosciences, UK) using 2 μl of anti-IFN-γ (1-D1K, FITC, MabTech). Absolute counts were determined using TRUcount™ tubes (BD Biosciences, UK) according to manufacturer's instructions. Clustered cells were determined by plotting forward scatter height (FSC-H) and intensity (FSC-A) and selecting events with large ‘time-of-flight’ (FSC-H) compared to intensity (FSC-A). All samples for flow cytometric analysis were run on a FACS Canto II (BD Biosciences™, UK) and analysis was performed on FACS Diva™ software (BD Biosciences™, UK). The gating strategy is described in the supplemental data (Figure S2).

Cytometric bead array (CBA) flex sets for human Macrophage Chemotactic Protein-1 (MCP-1), Interleukin-1β (IL-1β), Interleukin-6 (IL-6) and Transforming growth factor-β (TGF-β) were used in accordance with manufacturer's instructions (BD Biosciences™, UK). Additionally, a marmoset-compatible IFN-γ CBA was generated using antibodies supplied by MabTech. In short, unconjugated CBA bead E4 (BD Biosciences™, UK) was conjugated to the anti-human IFN-γ specific, biotinylated antibody B6-1 (MabTech, Sweden) using the bead conjugation kit according to manufacturer's instructions (BD Biosciences™, UK). Interferon concentration was than elucidated using the anti-human IFN-γ specific antibody D1K conjugated to FITC (MabTech™, Sweden) and a human interferon-γ standard (BD Biosciences™, UK) and samples were analysed as above.

For histopathology, tissues were fixed in 10% buffered formalin solution immediately following excision and processed for paraffin wax embedding using standard techniques. Sections (5±2 µm) were cut and stained with haematoxylin and eosin (H&E). Slides were randomised and sent for pathological scoring at the Animal Health and Veterinary Laboratory Agency (AHVLA, Weighbridge, UK).

### Re-stimulation of splenocytes

Splenocytes were reconstituted to 1×10^6^ cells/ml of RPMI medium with 10% foetal bovine serum (Gibco), glutamine (10 mM), penicillin (200 U/ml) and streptomycin (200 μg/ml) (Sigma-Aldrich™). 100 μl of cell suspensions were transferred to a 96 well tray and co-incubated with either 1 μl of PBS, 1 μl heat killed *B. pseudomallei* strain K96423 (a final concentration of 10 μg of protein/ml, from the Porton Down strain collection) or 0.6 μl of CHDMAPP in 1 μl of PBS. 1 μl of Golgi plug (Becton Dickinson™) was added and the cells incubated for 6 h at 37°C in a CO_2_ rich atmosphere. Samples were analysed by flow cytometry as described above.

### Bacterial culture

In this study we used *Burkholderia pseudomallei* strain NCTC 13392 [22]. Bacteria were cultured in 100 ml of Luria Broth (Gibco), in 250 ml conical flasks and incubated at 37°C on a rotary platform at 150–200 rpm overnight. Bacterial suspensions were diluted to a specific optical density (OD_590 nm_) with a known viable count, which was then diluted into PBS for challenge. Bacterial suspensions were enumerated by serial dilution in 24-well trays and plating on Luria agar. Bacterial colonies were counted after 1–2 days of incubation at 37°C.

### Statistical analysis

Graphs and analysis were performed using Graphpad PRISM V4.0. All analysis was performed using the statistical program IBM SPSS version 18.0. All data analysed in this study, with the exception of the pathology scores, was found to be exponential in nature and were log transformed prior to parametric analysis. Repeated measures univariate general linear models were the main analysis method where the repeated measures of individual marmosets were taken as the basis of repeated measures (time or organ). Bonferroni's post tests were performed for more detailed analysis. Spearman's correlations were performed for the liver pathology scores and Pearson's correlations were performed for all other analysis.

## Supporting Information

Figure S1 **A full correlation matrix of the interactions between several parameters observed in marmosets pre-treated with CHMDAPP+IL-2 and infected with** ***B. pseudomallei*** **.** Animals were treated with CHMDAPP and IL-2 (n = 7), and group treated with IL-2 only (n = 7). Animals received a single dose of CHMDAPP (day 0, at 2.5 mg/kg) and five doses of IL-2 (days 0, 1, 2, 3, 4 and 5, at 0.18 U×10^6^ per kg) or only received the five doses of IL-2 or received PBS only. Animals were then challenged with 77–1,081 CFU of *B. pseudomallei* strain K96423 at day 5 post onset of treatment. Multiple interactions were investigated and Pearson's correlations were performed with the exception of correlation to liver pathology scores, where the Spearman's method was used. Significant positive correlations are marked in orange and significant negative in blue.
(TIF)

Figure S2 **The gating strategy for the analysis of marmoset flow-cytometry samples.** One example sample is showed with gates above and the population hierarchy below.
(TIF)

Data S1 **A description of the interactions between several parameters observed in marmosets pre-treated with CHMDAPP+IL-2 and infected with** ***B. pseudomallei*** **.** Animals were treated with CHMDAPP and IL-2 (n = 7), and group treated with IL-2 only (n = 7). Animals received a single dose of CHMDAPP (day 0, at 2.5 mg/kg) and five doses of IL-2 (days 0, 1, 2, 3, 4 and 5, at 0.18 U×10^6^ per kg) or only received the five doses of IL-2 or received PBS only. Animals were then challenged with 77–1,081 CFU of *B. pseudomallei* strain K96423 at day 5 post onset of treatment. Multiple interactions were investigated and Pearson's correlations were performed with the exception of correlation to liver pathology scores, where the Spearman's method was used.
(DOCX)
