# Supplementary figures and images for: In Vivo Manipulation of γ9+ T Cells in the Common Marmoset (Callithrix Jacchus) with Phosphoantigen and Effect on the Progression of Respiratory Melioidosis
Source: PLoS One. 2013 Sep 30;8(9):e74789. doi: 10.1371/journal.pone.0074789 (PMC3786980; doi:10.1371/journal.pone.0074789)

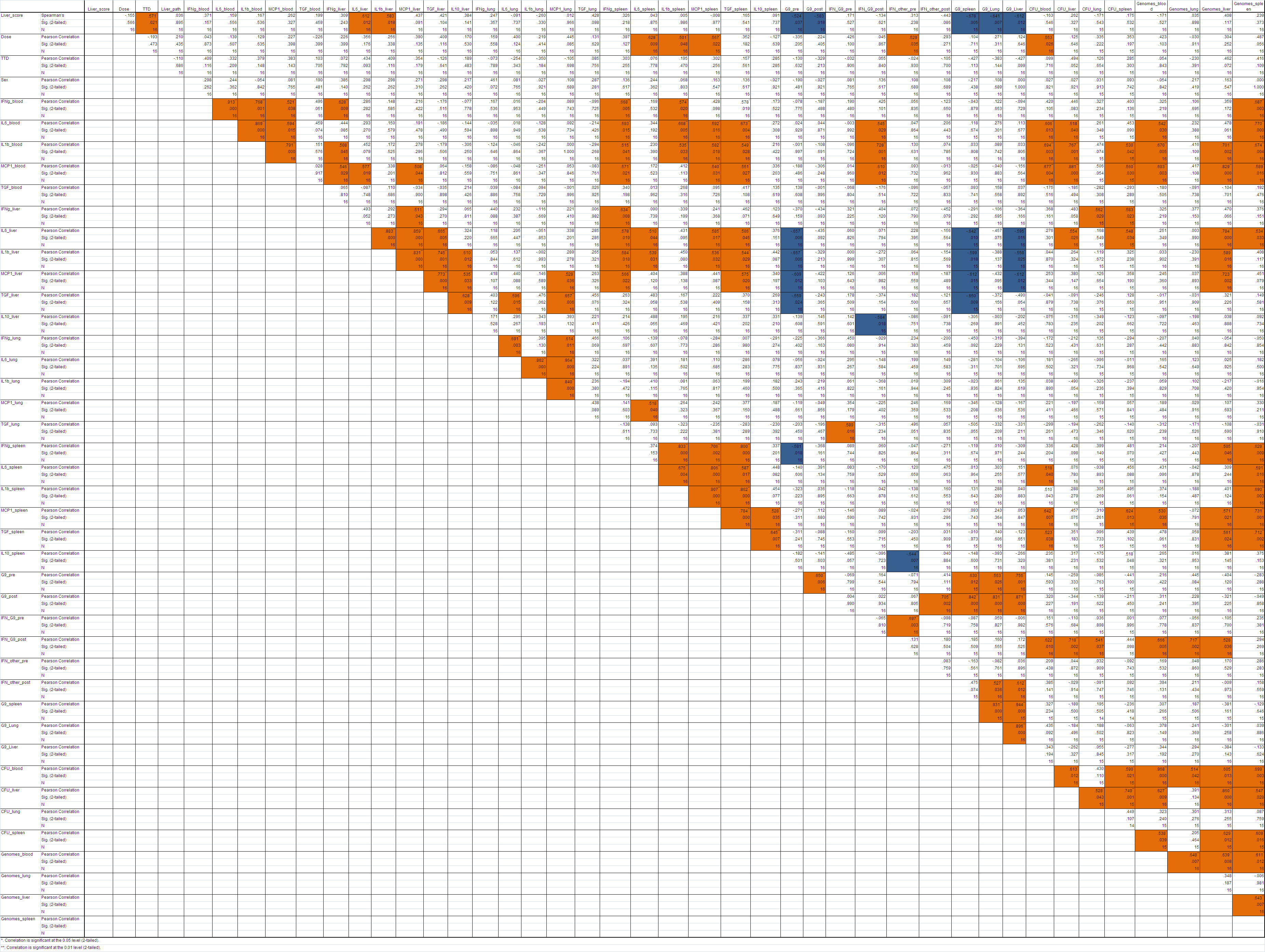

Supplement: Figure S1 — A full correlation matrix of the interactions between several parameters observed in marmosets pre-treated with CHMDAPP+IL-2 and infected with B. pseudomallei . Animals were treated with CHMDAPP and IL-2 (n = 7), and group treated with IL-2 only (n = 7). Animals received a single dose of CHMDAPP (day 0, at 2.5 mg/kg) and five doses of IL-2 (days 0, 1, 2, 3, 4 and 5, at 0.18 U×106 per kg) or only received the five doses of IL-2 or received PBS only. Animals were then challenged with 77–1,081 CFU of B. pseudomallei strain K96423 at day 5 post onset of treatment. Multiple interactions were investigated and Pearson's correlations were performed with the exception of correlation to liver pathology scores, where the Spearman's method was used. Significant positive correlations are marked in orange and significant negative in blue. (TIF) [file pone.0074789.s001.tif]

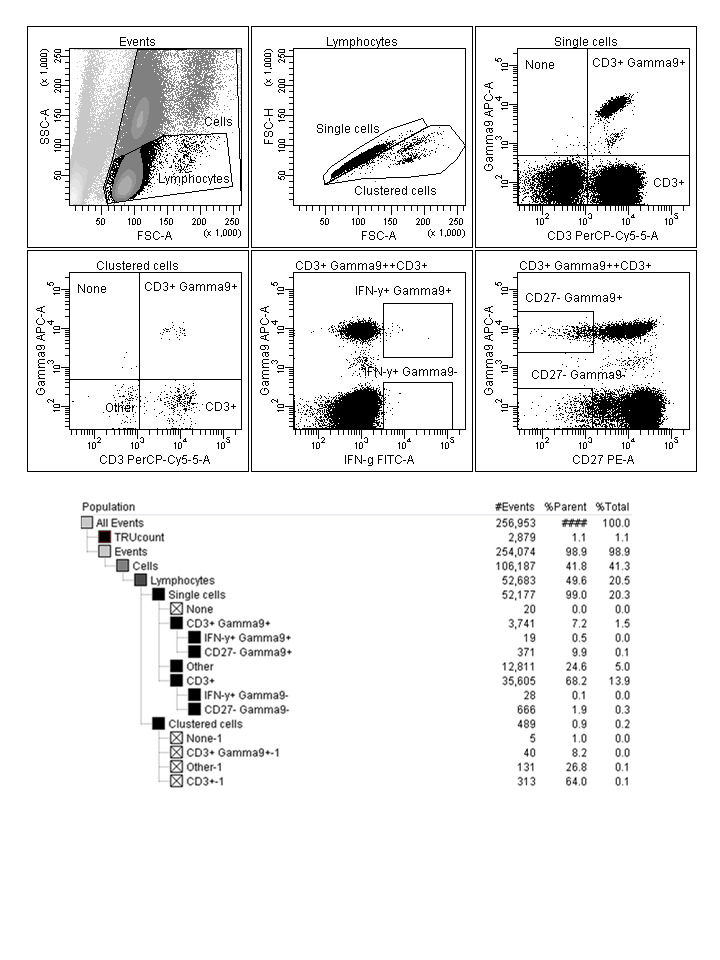

Supplement: Figure S2 — The gating strategy for the analysis of marmoset flow-cytometry samples. One example sample is showed with gates above and the population hierarchy below. (TIF) [file pone.0074789.s002.tif]
