# Supplementary material for: In Vivo Manipulation of γ9+ T Cells in the Common Marmoset (Callithrix Jacchus) with Phosphoantigen and Effect on the Progression of Respiratory Melioidosis
Source: PLoS One. 2013 Sep 30;8(9):e74789. doi: 10.1371/journal.pone.0074789 (PMC3786980; doi:10.1371/journal.pone.0074789)
Supplement: Data S1 — A description of the interactions between several parameters observed in marmosets pre-treated with CHMDAPP+IL-2 and infected with B. pseudomallei . Animals were treated with CHMDAPP and IL-2 (n = 7), and group treated with IL-2 only (n = 7). Animals received a single dose of CHMDAPP (day 0, at 2.5 mg/kg) and five doses of IL-2 (days 0, 1, 2, 3, 4 and 5, at 0.18 U×106 per kg) or only received the five doses of IL-2 or received PBS only. Animals were then challenged with 77–1,081 CFU of B. pseudomallei strain K96423 at day 5 post onset of treatment. Multiple interactions were investigated and Pearson's correlations were performed with the exception of correlation to liver pathology scores, where the Spearman's method was used. (DOCX) [file pone.0074789.s003.docx]

Supporting data

# In vivo Manipulation of γ9+ T cells in the Common Marmoset (*Callithrix Jacchus*) with Phosphoantigen and effect on the Progression of Respiratory Melioidosis.

Thomas R. Laws, Michelle Nelson, Cecile Bonnafous, Helene Sicard, Christopher Taylor, Francisco Javier Salguero, Timothy P. Atkins, Petra C. F. Oyston, Caroline A. Rowland.

# Correlations

All parameters that were measured at the lethal end point for the animals in the *B. pseudomallei* experiments were subjected to correlation analysis (Figure S1). In short these animals were treated with either CHMDAPP and IL-2 (n = 7) or with IL-2 only (n = 7). Animals received a single dose of CHMDAPP (day 0, at 2.5 mg/kg) and five doses of IL-2 (days 0, 1, 2, 3, 4 and 5, at 0.18 U x 10^6^ per kg). Animals were then challenged with 77-1,081 CFU of *B. pseudomallei* strain K96423 at day 5 post onset of treatment. Spearman’s or Pearson’s correlation were performed as appropriate to evaluate the likelihood of a relationship between each parameter.

One concern in using our approach to identify correlates of factors measured within this experiment is that there may not be homogeneity between treatment groups and each group might show different correlations. This concern was addressed by performing correlations for both the CHDMAPP and IL-2 treated and IL-2 only treated groups individually. The differential of the two derived correlation values was found to have a normal distribution with a mean of close to 0. A one sample T-test was applied to these differential values and we found that it would be highly unlikely that the experimental readouts for each treatment groups correlated differently (data not shown).

Several significant correlations we found that were associated with the ‘direct health readouts’ of the animals (Liver pathology and time-to-death) (Figure 8). The only correlation observed to time to death was liver pathology (Spearman’s R = 0.571, P = 0.021). Liver pathology also correlated positively with concentrations of IL-6 (Spearman’s R = 0.612, P = 0.012) and IL-1β (Spearman’s R = 0.583, P = 0.018) within the liver. Also, liver pathology negatively correlated with the proportion of T cells γ9 within the blood pre-infection (Spearman’s R = -0.524, P = 0.037) and post mortem (Spearman’s R = -0.583, P = 0.018) and in the lung (Spearman’s R = -0.641, P = 0.005), liver (Spearman’s R = -0.612, P = 0.012) and spleen (Spearman’s R = -0.678, P = 0.005) post mortem.

Regarding the concentration of γ9 T cells in the blood prior to infection; we found negative correlations to liver IL-6 (Pearson’s R = -0.657, P = 0.006), liver IL-1β (Pearson’s R = -0.657, P = 0.006), liver MCP-1 (Pearson’s R = -0.609, P = 0.012) and liver TGF-β (Pearson’s R = -0.559, P = 0.024) and IFN-γ in the spleen (Pearson’s R = -0.581, P = 0.018). We found positive correlations between the concentration of γ9^+^ T cells in the blood prior to infection and their concentration post mortem in the blood (Pearson’s R = 0.650, P = 0.06), lung (Pearson’s R = 0.553, P = 0.026), liver (Pearson’s R = 0.755, P = 0.001) and spleen (Pearson’s R = 0.630, P = 0.012). The concentration of γ9^+^ T cells in the blood post mortem correlated to the proportion of γ9^-^ T cells IFN-γ^+^ (Pearson’s R = 0.705, P = 0.002), and the concentrations of γ9 T cells in the lung (Pearson’s R = 0.831, P < 0.001), liver (Pearson’s R = 0.871, P < 0.001) and spleen (Pearson’s R = 0.842, P < 0.001) post mortem. The concentration of lung γ9^+^ T cells correlated with the proportions IFN-γ^+^ producing γ9^-^ T cells in the blood post mortem (Pearson’s R = 0.527, P = 0.036) and the concentration of γ9^+^ T cells in the liver (Pearson’s R = 0.896, P < 0.001) and spleen (Pearson’s R = 0.931, P < 0.001). The concentration of liver γ9^+^ T cells correlated with the proportions IFN-γ^+^ producing γ9^-^ T cells in the blood post mortem (Pearson’s R = 0.612, P = 0.012) and the concentrations of γ9 T cells in the spleen (Pearson’s R = 0.896, P < 0.001). The concentration of liver γ9 T cells negatively correlated with the concentration of liver IL-6 (Pearson’s R = -0.595, P = 0.015), IL-1β (Pearson’s R = -0.558, P = 0.025) and MCP-1 (Pearson’s R = -0.612, P = -0.012). The concentration of spleen γ9^+^ T cells negatively correlated with the concentration of liver IL-6 (Pearson’s R = -0.642, P = 0.010), IL-1β (Pearson’s R = -0.599, P = 0.018), MCP-1 (Pearson’s R = -0.612, P = 0.015) and TGF-β (Pearson’s R = -0.650, P = 0.009).

The proportion of IFN-γ^+^ γ9 T cells prior to infection correlated with TGF-β in the lung (Pearson’s R = 0.589, P = 0.016) and IFN-γ^+^ γ9^-^ T cells (Pearson’s R = 0.697, P = 0.003). The proportion of IFN-γ^+^ γ9^+^ T cells post mortem correlated with blood IL-6 (Pearson’s R = 0.545, P = 0.029), IL-1β (Pearson’s R = 0.728, P = 0.001) and MCP-1 (Pearson’s R = 0.610, P = 0.012) and to IFN-γ^+^ other T cells (Pearson’s R = 0.705, P = 0.002). The proportion of IFN-γ^+^ γ9^+^ T cells prior to infection negatively correlated with IL-10 in the liver (Pearson’s R = -0.644, P = 0.007). The proportion of IFN-γ^+^ γ9^-^ T cells prior to infection correlated with estimated challenge dose (Pearson’s R = 0.529, P = 0.035) and negatively correlated with splenic IL-10 (Pearson’s R = -0.644, P = 0.007). The proportion of IFN-γ^+^ γ9^-^ T cells post mortem correlated with viable bacteria in the blood (Pearson’s R = 0.622, P = 0.010), liver (Pearson’s R = 0.718, P = 0.002) and lung (Pearson’s R = 0.541, P = 0.037) and bacterial genomes in the blood (Pearson’s R = 0.666, P = 0.005), liver (Pearson’s R = 0.528, P = 0.036) and lung (Pearson’s R = 0.717, P = 0.002).

We found correlations to the initial estimated challenge dose. Challenge dose correlated with splenic IL-6 (Pearson’s R = 0.628, P = 0.009), IL-1β (Pearson’s R = 0.501, P = 0.048), MCP-1 (Pearson’s R = 0.567, P = 0.022) and culturable bacteria in the blood (Pearson’s R = 0.553, P = 0.026), all post mortem.
